# Proposal for a New Noncontact Method for Measuring Tongue Moisture to Assist in Tongue Diagnosis and Development of the Tongue Image Analyzing System, Which Can Separately Record the Gloss Components of the Tongue

**DOI:** 10.1155/2015/249609

**Published:** 2015-01-28

**Authors:** Toshiya Nakaguchi, Kanako Takeda, Yuya Ishikawa, Takeshi Oji, Satoshi Yamamoto, Norimichi Tsumura, Keigo Ueda, Koichi Nagamine, Takao Namiki, Yoichi Miyake

**Affiliations:** ^1^Center for Frontier Medical Engineering, Chiba University, 1-33 Yayoi-cho, Inage-ku, Chiba 263-8522, Japan; ^2^Graduate School of Engineering, Chiba University, 1-33 Yayoi-cho, Inage-ku, Chiba 263-8522, Japan; ^3^Graduate School of Advanced Integration Science, Chiba University, 1-33 Yayoi-cho, Inage-ku, Chiba 263-8522, Japan; ^4^Department of Japanese-Oriental (Kampo) Medicine, Graduate School of Medicine, Chiba University, 1-8-1 Inohana, Chuo-ku, Chiba 260-8670, Japan; ^5^Akiba Clinic of Traditional Medicine, Ni-2086 Hasunuma, Sanmu-shi, Chiba 289-1805, Japan

## Abstract

Tongue diagnosis is a noninvasive diagnosis and is traditionally one of the most important tools for physicians who practice Kampo (traditional Japanese) medicine. However, it is a subjective process, and its results can depend on the experience of the physician performing it. Previous studies have reported how to measure and evaluate the shape and color of the tongue objectively. Therefore, this study focused on the glossy component in order to quantify tongue moisture in tongue diagnosis. We hypothesized that moisture appears as a gloss in captured images and measured the amount of water on the tongue surface in 13 subjects. The results showed a high correlation between the degree of gloss and the amount of water on the tongue surface and suggested that the moisture on the tongue can be estimated by the degree of gloss in a captured image. Because the moisture level on the tongue changes during the course of taking photos, it became clear that we had to wait at least 3 minutes between photos. Based on these results, we established the tongue image analyzing system (TIAS), which can consistently record the gloss and color of the tongue surface simultaneously.

## 1. Introduction

Tongue diagnosis is one of the most important diagnostic methods for physicians who practice Kampo medicine. We use tongue diagnosis to determine a patient's condition and the progress of a disease by thoroughly observing the quality of and the coat on the dorsum and underside of the tongue. We perform tongue diagnosis by observing the color and moisture of the tongue surface, the shape of the tongue, and the characteristics of the coating on the tongue surface. Many studies have reported a correlation between the shape and color of the tongue and an individual's health [1, 2]. In addition, it has been found that the moisture of the tongue reflects the water metabolism in the body [3]. In other words, a high degree of tongue moisture may reflect an abnormality in the water metabolism in the body, which may signify some type of dysfunction in the internal organs. On the other hand, when the degree of tongue moisture is low and the tongue is dry, we suspect that the patient suffers from dehydration and heat production. Thus, with tongue diagnosis, we can noninvasively diagnose the state of the blood and humors of the whole body, the autonomic nervous system, and especially the upper gastrointestinal tract. However, tongue diagnosis depends on the experience and subjective opinion of the physician, and the diagnostic results are qualitative and inconsistent. In addition, external environmental factors such as the type and direction of the lighting affect the appearance of the tongue.

In order to solve these problems, many researchers have developed computer tongue diagnostic support systems that use image processing [4–9]. However, with these techniques, gloss on the tongue is visible when the image is captured, and the systems cannot correctly obtain the color of the portion of the tongue masked by the gloss. For example, when the blood circulation stagnates, small purple spots may appear on the surface of the tongue. As the glossy area will change in response to slight movements of the tongue and to changes in the physician's line of sight, the reflective area is not fixed, and spots hidden by the reflective area will eventually be revealed through movement. In contrast, a systematized imaging environment prioritizes stability in general. It fixes the camera and subject positions and also limits the number of pictures taken, increasing the risk that spots will be masked by the gloss and will be overlooked. In tongue diagnosis in the practice of Kampo medicine, tongue moisture needs to be observed separately from the shape and color of the tongue.

In order to measure the moisture of the oral submucosa objectively, an electronic device named Mucus III (Life Co., Ltd.) was developed [10–12]. Since this device is based on the concept of the electronic condenser, it needs the probe of the device be pressed against the mucosal surface of the tongue for five to ten seconds while applying constant pressure of 200 g/cm^2^. This is actually not easy for the operator to control the pressure, and furthermore it is unacceptable for some people that such unfamiliar device directly touches their tongue for long time. To address such problems, noncontact and shorter-time measurement method is highly required.

When the dorsum of the tongue is observed, the exposed part that protrudes from the mouth generally runs from the terminal sulcus to the proglossis. The lingual mucosa of the tongue surface is composed of tissues that are concave or convex, such as the median groove and the tongue papilla. Furthermore, since each tissue scatters light internally, if the tongue surface is dry, the tissue does not generate the gloss caused by the reflection of incident light. However, when powerful directed lighting illuminates the surface of the tongue body and a picture is taken, we generally observe a great deal of gloss. It is thought that liquid such as saliva adheres to the surface of the tongue body and covers the median groove and irregular parts of the lingual papillae, and the surface reflection on the liquid causes the gloss. Therefore, it is thought that the degree of gloss recorded with the camera is correlated with the amount of water that adheres to the surface of the tongue body. In order to analyze the correlation between the degree of gloss and the moisture of the tongue surface, a technique that can measure the degree of gloss on the tongue surface consistently and quantitatively is needed. However, detailed studies have not been performed up to now; here we investigate a quantitative method for measuring the degree of gloss on the tongue surface.

First, we examine which of the three imaging methods (45-degree lighting, polarizing plates, and integrating sphere) is best for controlling the gloss. This examination will enable us to take pictures of the tongue independent of the surrounding lighting environment. Next, we will examine noncontact and quantitative methods of measuring tongue moisture to extend our ability to record the condition of the tongue. We hypothesize that tongue moisture appears in images as gloss. First, we examine a method for measuring the gloss consistently. In order to record gloss, we record the specular reflection light with a camera by irradiating light that is strongly directed at the tongue surface. We need to determine suitable geometry for the lighting and to identify a camera that can record the gloss consistently. The third objective is to examine the method for calculating the degree of gloss from pictures and measuring the volume of water on the tongue surface. We then analyze the correlation between the degree of gloss and the tongue moisture. In addition, we evaluate the accuracy with which the tongue moisture was measured based on the degree of gloss. The fourth objective is to evaluate the recovery time needed for the tongue to recover its moisture. This evaluation is important for determining the duration to wait between taking photographs when repeatedly capturing images. Finally, we develop a new tongue image analyzing system (TIAS) that can separately record the gloss portion and nongloss portion of the image.

This research was carried out in accordance with the Chiba University Graduate School of Medical Studies Ethical Review Board number 812.

## 2. Method

### 2.1. Evaluation of Image Geometry That Controls the Gloss of the Tongue

The surface of an object is observed through a combination of surface-reflected light and internally reflected light based on a dichromatic reflection model. When observing a tongue surface, although the color of the tongue and the coating of the tongue appear as internally reflected light, the part to which liquids such as saliva adhere will radiate strong surface-reflected light (i.e., gloss) depending on the angle of the lighting. When the lighting direction and the observation direction are such that specular reflection is created, the surface-reflected light becomes very strong compared to the internally reflected light, and the internally reflected light is masked and cannot be observed. Therefore, in order to observe the color and coating of the tongue, a photographic method that can control gloss is needed. Generally, there are three kinds of measurement methods that can control gloss. The first involves using 45-degree lighting; the lighting is arranged so that light enters at an angle of 45 degrees to the normal line of a reflective surface and suppresses the specular reflecting light when observed from the normal (0°) direction. The second uses a set of polarizers; the angle of the polarizer in front of the lighting source and that of the polarizer in front of the camera differ by 90°. The third uses diffuse illumination within an integrating sphere. By photographing the dorsum of the tongue using the above three methods to suppress gloss, we can compare and evaluate which method is best for photographing the dorsum of the tongue in terms of the degree of gloss inhibition and the quality of the pictures.

### 2.2. Development of TIAS

**Figure 1:**
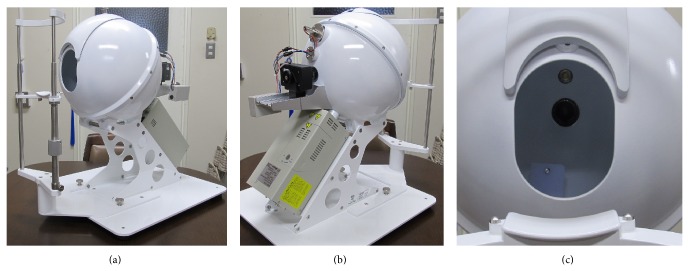
The appearance of the tongue image analyzing system (TIAS). (a) Front side: aperture and jaw stand. (b) Back side: camera and high direct light source. (c) Inside appearance of the integrating sphere from the opening.

We developed TIAS equipped with a diffused light source for recording the state of a tongue surface. The TIAS is shown in Figure 1. The inside diameter of the integrating sphere is 300 mm, and the inner wall is uniformly painted with barium sulfate. The area of the opening is about 7800 mm^2^, and the shape is slightly elliptical so that it can fit into the mouth with the mouth open. Moreover, the system can be used to take a child's image; it is possible to reduce the size of the opening with a slide system cover. When the cover is closed, it also functions as internal protection for the integrating sphere when not in use. The camera (Lumenera Lw115 C-1280 × 1024-pixel color CMOS sensor) was set directly facing the opening. The camera was equipped with a lens with a focal length of 16 mm. A halogen light source (Moritex MHAB-150 W, color temperature 3200 K) of 150 W was installed in the lower part of the rear surface of the integrating sphere as a light source for diffuse illuminations. A baffle plate was installed so that this light source would not directly illuminate the photographic subject. In order to circulate the air inside the integrating sphere, air is evacuated through the hole used for light sources. Moreover, the jaw stand is movable from front to back and up and down, and it can be adjusted to a suitable position according to the subject.

In order to calibrate the characteristic changes of the camera or light source, calibration is performed only once when the power is turned on. For calibration, color charts (X-rite Color Checker Passport) of 24 colors are installed in the integrating sphere opening, an exposure is taken, and the color gain of the camera is adjusted automatically. The shutter speed of the camera is fixed at 20 ms. When using TIAS to take images of a subject, 10 tongue images are taken in 1 second at intervals of 0.1 seconds. The images are saved sequentially as lossless 24 bit RGB format color images. Then the RGB values are converted to CIE1976 L^*^a^*^b^*^ color space via XYZ color space. The conversion matrix from RGB to XYZ color space is estimated from the color charts by using the multiple regression method.

### 2.3. Measurement of Gloss

**Figure 2:**
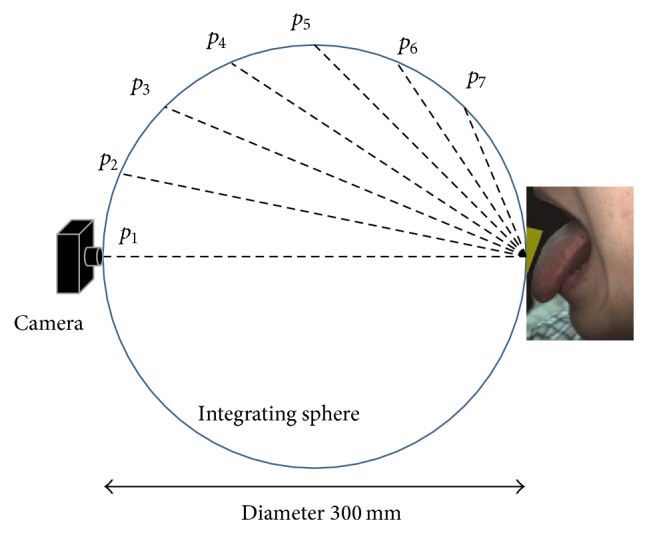
Placement of camera and LED lighting to determine the best photographic geometry.

In order to determine the best placement for achieving suitable imaging geometry as shown in Figure 2, the camera was set at the front of the face. It was arranged so that the position of the LED lighting can change from *p*_1_ (zero ascending vertical angle) to *p*_7_ (67.5 ascending vertical angles) in increments of 11.25 degrees. In addition, since the system is premised on using for measuring gloss together with consistent measurement of tongue color, LED lighting was installed in the inner wall of the 300 mm diameter integrating sphere. Moreover, the LED was attached to a focusing lens with a 10-degree angle spread. In this research, we compared and examined the optimal LED position for measuring gloss clearly and consistently.

### 2.4. Analysis of Correlation between the Amount of Moisture on the Tongue Surface and Gloss Degree

In order to quantify the degree of gloss from the images taken by the camera, two indices, gloss luminosity and glossy area, were considered. Since gloss is a specular reflection component of the illumination lights, a camera with a very high dynamic range is required in order to measure the gloss luminosity. However, high-dynamic-range cameras are expensive, specialty items that are not generally used. In this system, we measure the glossy area as an index of the degree of gloss on the assumption that a general-purpose industrial camera can be used to promote the spread of the system in a lower price range. In order to measure the gloss, it is sufficient to capture an image under lighting with high directivity. However, since the diffuse illumination light source for color measurement cannot be turned on and off frequently, the gloss must be photographed with diffuse illumination at all times. Therefore, in this study, by taking two images, one nonglossy image taken by irradiating only with diffuse illumination and another glossy image taken by irradiating with both a strong directional light source and diffuse illumination, the gloss component is detected by the following procedure. First the nonglossy image is subtracted from the glossy image. Since pixel values in the subtraction image can clearly be classified into two groups, one is gloss and another is dark noise, we obtain the glossy area by applying Otsu's thresholding method [13]. It is ideal to take the two pictures simultaneously in order to perform the picture subtraction, but since capturing simultaneous images by light separation greatly complicates the system configuration, we took two images in succession in a short time. The index of the glossy area was computed as the ratio of the area of the glossy part to the area of the whole surface of the tongue expressed as a percentage.

**Figure 3:**
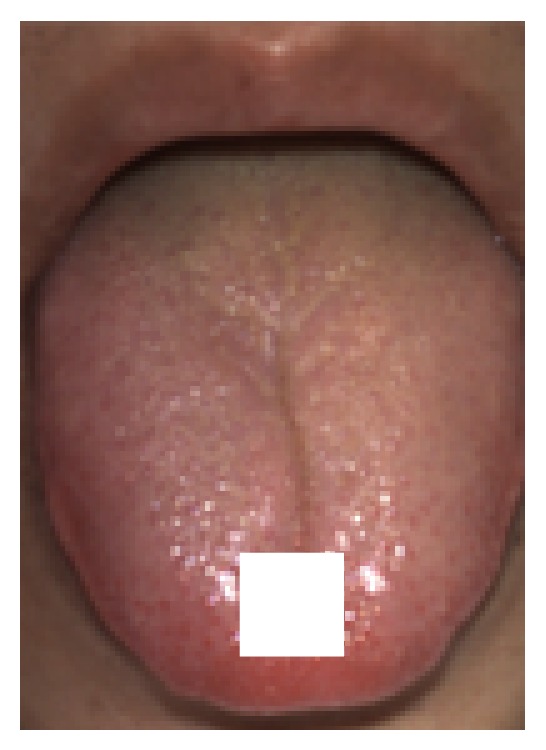
Filter paper used to measure the moisture on the tongue surface; a 10 mm square filter paper was placed at the center and 10 mm from the tip of the tongue.

The next process was to measure the amount of moisture by extracting moisture from the tongue surface and measuring the weight of water gain. To select a material that extracts moisture from the tongue surface effectively, we compared filter paper, gauze, kitchen paper, and tissue paper. Among them, kitchen paper and tissue paper were easy to be torn and folded and very hard to handle; they were excluded from the candidates. Then we compared the amount of absorbed moisture under the controlled condition, and the filter paper (56 mg) absorbed three times larger than the gauze (17 mg). Based on the moisture absorbency and the ease of handling, filter paper was found to be the most suitable material. The procedure for measuring the amount of tongue surface moisture was as follows: filter paper cut into 10 mm squares and a sealing bag were weighed in advance using an electronic scale.  Figure 3 shows the measurement in which 10 mm squares of filter paper were placed on the center line 10 mm from the tip of the subject's tongue for five seconds. This measurement position is determined by referring to the previous work [10]. At this time, the exact position of the filter paper was recorded by a picture for the image measurement. After five seconds, the filter paper was removed from the tongue and put into the sealing bag so that it would not lose any of the moisture that it had absorbed, and the weight of the filter paper was measured. The amount of moisture was calculated by deducting the weight of the filter paper and the sealing bag from the measured value.

Measurements were carried out on 15 subjects (8 males, 7 females) in two states: a normal state and another in which the tongue surface was thoroughly dried. Immediately after gloss and nongloss images were captured one by one using TIAS, the amount of water on the tongue surface was measured. Since the image is recorded in an instant, the tongue surface did not dry at all during the measurement. In the images captured using TIAS, the gloss component was determined from the difference between the gloss image taken using the LED light source and the nongloss image taken with the LED light source switched off. The area where the filter paper was placed on the tongue surface for the measurement of moisture was manually specified as the area of interest referring to the picture of the filter paper on the tongue, and the degree of gloss of the area of interest was computed. The average RGB value of the area of interest in each picture was converted into a CIE1976 L^*^a^*^b^*^ color space, and the brightness (L^*^ value) of the picture was calculated. The difference in the brightness value of the area of interest between the nongloss picture and the gloss picture was calculated as the degree of gloss.

### 2.5. Examination of the Moisture Recovery Time of the Dry Tongue Surface

When repeating and carrying out moisture measurements on the tongue surface, we examined the recovery time and the degree of recovery of the moisture on the tongue surface between photos. Images were captured repeatedly to measure the serial change in the degree of gloss when the tongue was stuck out for 20 seconds. The procedure was then repeated after an interval, which varied from 10 seconds to four minutes.

## 3. Results

### 3.1. Examination of the Image Geometry for Suppressing Gloss

**Figure 4:**
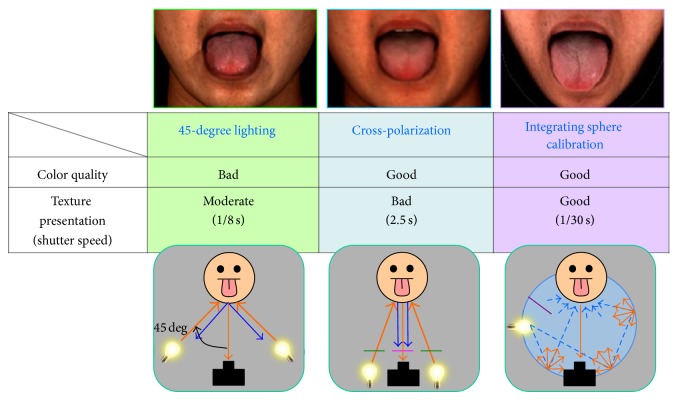
Comparison of three methods to capture nonglossy tongue image.

Figure 4 shows the results of the application of three kinds of imaging methods to observe the tongue surface. It was found that the 45-degree lighting could not fully suppress the surface reflection light and gloss was partially recorded. Polarizer imaging suppressed the gloss sufficiently, but the weakness of the light intensity made a longer exposure necessary, and as a result, the sharpness of the picture decreased. Generally, since the tongue cannot be immobilized, it is desirable for the exposure time to be short. The integrating sphere controlled the gloss satisfactorily, and the sharpness of the picture was high. Thus, we concluded that the integrating sphere lighting was most suitable for recording the character of a tongue and the color of the tongue coating.

### 3.2. Measurement of Gloss

**Figure 5:**
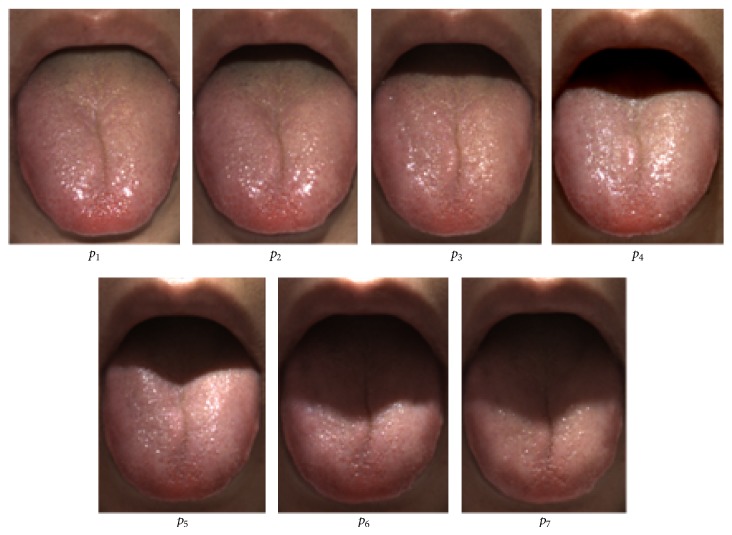
Shooting results with the arrangement of LED lighting from* p*_1_ to* p*_7_.

Figure 5 shows the results of photography with an LED lighting arrangement from *p*_1_ to *p*_7_. The gloss was recorded properly from *p*_2_ to *p*_4_. On the other hand, when the ascending vertical angle of the LED became large, a shadow of the upper lip appeared and blocked the posterior part of the tongue. Therefore, we determined the optimal arrangement angle of the LED to be 10 degrees in order to record a wide area including the posterior part of the tongue.

### 3.3. Extension of Function in TIAS

**Figure 6:**
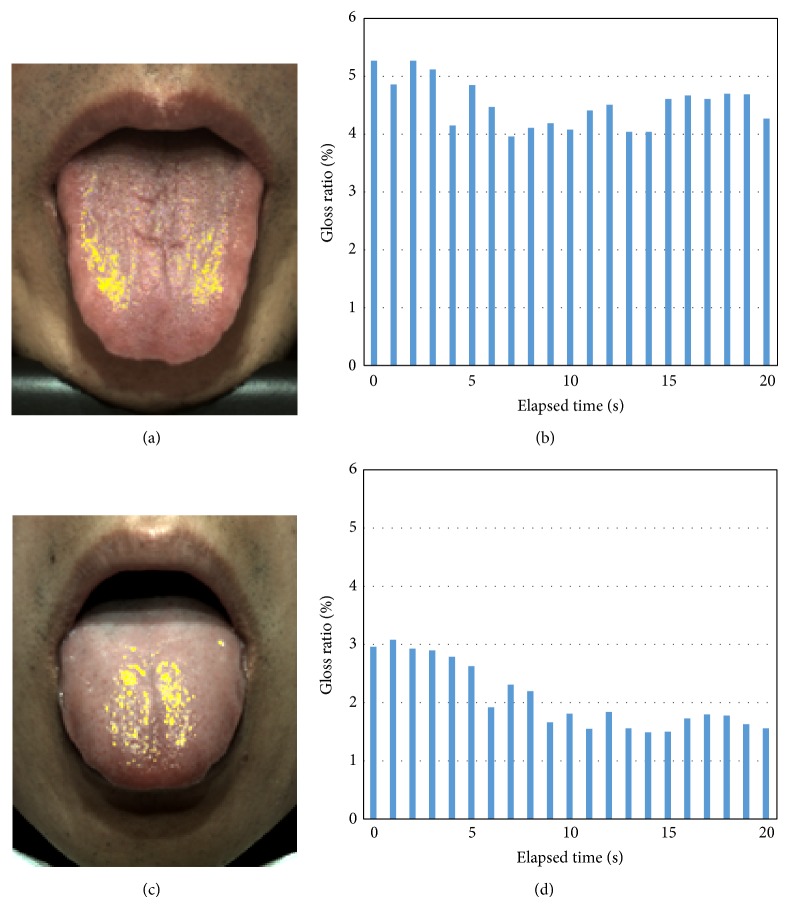
Serial change of the gloss ratio measured by TIAS. (a), (b) Subject 1. (c), (d) Subject 2. (a), (c) Gloss component shown as yellow area. (b), (d) Gloss ratio change over 20 seconds.

TIAS is enhanced by using the optimum arrangement of the high-directivity light source. The condenser lens of 10 degrees was attached to a high-intensity white LED (Luxeon LXK2-PWC4-0180, 180 lumen, color temperature 6500 K) as a high-directivity light source, and it was installed at a position 10 degrees above the subject. The microcomputer-controlled LED driving board was designed to enable on-off control of this high-directivity light source. The source of the diffused light is always set to on, and the LED light source is also turned on once per second; only one gloss picture is recorded. Since TIAS can shoot 10 pictures per second, nine color sheets of the lusterless tongue surface can be recorded in one second, and one sheet forms a picture that contains gloss. In order to calculate the degree of gloss, the difference between continuous gloss and nongloss pictures is used. The glossy area shown in Figures 6(a) and 6(c) can be obtained by the thresholding method from the subtraction image as described in Section 2.4. Figures 6(b) and 6(d) show the serial change of the degree of gloss as recorded by TIAS. Subject 1 had an approximately 5% degree of gloss immediately after sticking out his tongue; the amount fell to 4% after about 5 seconds and remained at about 4% for 20 seconds. On the other hand, subject 2 had a 3% degree of gloss immediately after the tongue was stuck out, and it decreased gradually over time and became 1.5% after 15 seconds. These results suggested that the degree of gloss varies by person immediately after the tongue is stuck out, and the rate of change of the degree of gloss after the tongue is stuck out also varies.

### 3.4. Analysis of Correlation between the Amount of Moisture on the Tongue Surface and the Degree of Gloss

**Figure 7:**
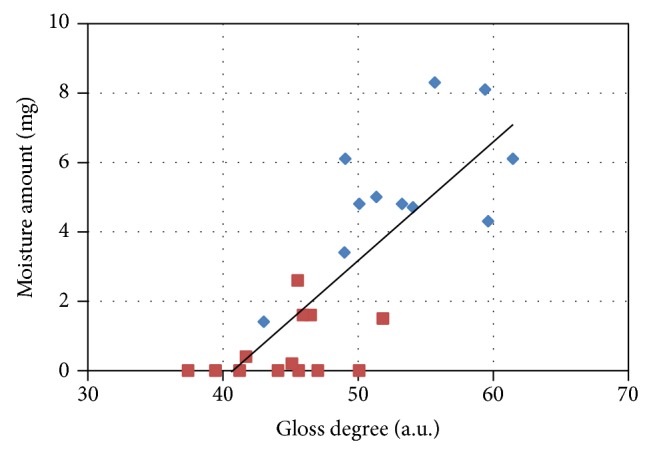
Relationship between the moisture amount and the degree of gloss on the tongue. Blue plot: normal condition. Red plot: dry condition.

We analyzed the photographic data for 12 subjects in a normal state and 13 subjects in a dry state. The cases in which the exposed tongue was nearly horizontal and the camera could not shoot it appropriately were excluded from analysis. Using the TIAS with enhanced functionality, the degree of gloss of the tongue surface was quantitatively measured, and the correlation between the amount of moisture on the tongue surface and the degree of gloss was analyzed.  Figure 7 shows the plot of the correlation between the amount of moisture on the tongue surface and the degree of gloss. The blue plots represent the 12 normal-state cases and the red plots represent the 13 cases in a dry state. This result revealed that the Pearson product-moment correlation coefficient of the amount of moisture on the tongue surface and the degree of gloss was 0.80, and a significant relationship was obtained at the 1% level. The regression formula, which presumes a moisture amount on the tongue surface based on the gloss degree measured in the picture, is as follows:

(1) Amount  of  moisture  mg  =4.2×10−4×gloss  degree−0.017.

### 3.5. Examination of the Moisture Recovery Time of the Dry Tongue Surface

**Figure 8:**
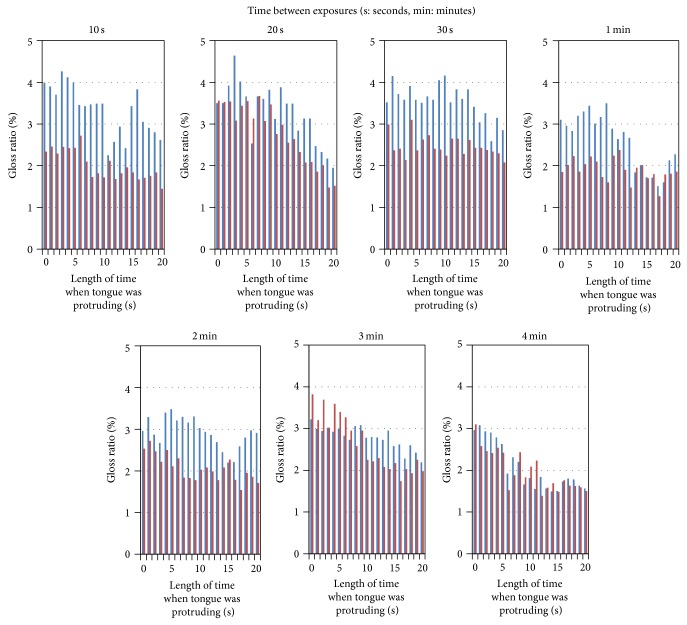
Degree of gloss on the tongue surface at each time point from 10 seconds to 4 minutes. Blue bar: gloss ratio in the first photo series. Red bar: gloss ratio in the second photo series.

Figure 8 shows the plot of the degree of moisture recovery of the tongue surface at different recovery times between image acquisitions. The blue plots in the first image series show that the degree of gloss decreased greatly in 20 seconds. In addition, when the recovery time between the acquisitions of images was set at 10 seconds, the moisture had not recovered, and the degree of gloss was decreased at the second image capture. This tendency continued up to a recovery time of two minutes; when the recovery time was set for three minutes or more, that gloss degree at the time of the second series had recovered to the same quantity seen in the first series. From the above results, it became clear that a period of three minutes or more was required for the recovery time between image acquisitions in order to repeatedly capture images for the purpose of stabilizing the measurement of the gloss degree.

## 4. Discussion

In this paper, we first examined several photographic techniques for controlling gloss in order to stabilize the color of the tongue surface and the coating of the tongue. We then developed TIAS using integrating sphere lighting. We explained our method of using polarizers with imaging techniques to control gloss, setting S polarization in front of the lighting and P polarization in front of the observation position. Only S polarized light struck the reflective surface, as the polarizing plate was placed before the lighting. Although surface reflection light was reflected with the state of S polarization maintained, internally reflected light became a mix of S polarization and P polarization due to the influence of dispersion. Only P polarization penetrated this reflection light, as the polarizing plate was placed in front of the observation position. This is a system in which the surface reflection light was intercepted and only some of the internally reflected light was observed. This method has the advantage that it can observe internally reflected light with high precision; however, the intensity of the observable light became weaker due to the polarizers. With diffuse illumination in an integrating sphere, the light emitted by the light source undergoes repeated diffuse reflection inside the sphere, and the reflective surface is uniformly illuminated from all directions. No specific specular reflection angle exists with diffuse illumination, and as a result we can observe internally reflected light without the masking effect of a glossy surface. Based on these principles, we developed a photographic device using an integration sphere.

We then investigated methods for measuring the tongue moisture quantitatively without contacting the tongue surface. It was shown that moisture is represented by the degree of gloss recorded in a picture, and we determined the imaging geometry with which the degree of gloss was measured stably. There was a high correlation of *R* = 0.8 between the degree of gloss and the water weight of the tongue surface as measured using TIAS, and it was suggested that the moisture amount on the tongue surface could be estimated from the degree of gloss. Although regression formula (1), which presumes an amount of moisture on the tongue surface based on the degree of gloss measured in an image, can have substantial quantitative error, determining the strict moisture weight of the tongue surface is not required in Eastern medicine. Since the amount of moisture is characterized on the basis of a three-to-four-degree scale ranging from “nothing” to “high,” we believe that regression formula (1) has sufficient accuracy to provide diagnostic support for Kampo medicine. As noncontact image-based measurement of the amount of moisture on the tongue surface becomes possible, it can be used for the diagnostic quantification of diseases such as Sjögren syndrome that present with abnormal amounts of moisture on the tongue surface and for determining the progress of medical treatment.

As this method enables us to visually identify the range and grade of dryness on a tongue surface, we expect to establish a quantitative evaluation method that differs from past methods. In order to raise the accuracy of the moisture amount estimated on the basis of the degree of gloss, it is necessary to normalize the data based on multiple measurements. The generation of gloss depends on the shape of tongue and condition of the saliva adhering to the tongue surface, on the shape of tongue and condition of saliva change based on the conditions in the mouth before the tongue is exposed, and on how the tongue is exposed. In order to control the variation in gloss measurement due to such changes, multiple measurements should be taken. However, as Figure 8 shows, when photographing the tongue in an exposed state for a definite period, the surface dryness and degree of gloss of the tongue decrease gradually. Therefore, in order to repeat the gloss measurement accurately, it is necessary that the tongue be kept in the mouth to recover its natural moisture. This study revealed that an interval of three minutes or more is required between measurements. Until now, the length of this interval could not be estimated; now, for the first time, this estimation is possible using the computer-based method for measuring the moisture on the tongue surface established in this study. The development of this method suggests the possibility of a new quantitative method of performing tongue diagnosis.

Furthermore, this study does not distinguish the type of saliva that generates gloss. Although the saliva on the tongue surface is a mixture of mucous and serous saliva, it is difficult to visually distinguish different types of saliva. On the other hand, it became clear that the dryness of the tongue surface varied among the subjects as they continued to hold their tongues out, as shown in Figure 6. If we hypothesize that there are differences in drying between mucous saliva and serous saliva, the mixture ratio of the saliva types may be detectable from the changing trend of the degree of gloss on the tongue surface as subjects continue to hold out their tongues. This will be a subject for future examination.

## 5. Conclusion

This study proposes a tongue imaging system called TIAS, which can establish tongue color quantitatively and consistently. Also, TIAS can record the color and gloss of the tongue surface separately. In order to raise the accuracy of the quantitative moisture estimates, it is necessary to obtain more consistent measurements of the degree of gloss. In addition, we have confirmed that the regression formula, which estimates the moisture from the degree of gloss, has sufficient accuracy to support moisture diagnosis in the performance of tongue diagnosis in Kampo medicine. Since the tongue surface dries and the moisture changes at every measurement, it is necessary to take careful, repeated measurements. This study examined the time required for the tongue to recover its moisture between repeated measurements, and it was found that an interval of more than three minutes was necessary. The method of measuring the moisture on the tongue surface using a computer is quantitative. Moreover, as this system measures a two-dimensional distribution of tongue color, it provides new information that was not available for tongue diagnosis in the past. We can expect a new method of tongue diagnosis using this measurement technique and other applications for tongue diagnosis to be established in further investigations.
